# Supplementary material for: Atomic structure and defect dynamics of monolayer lead iodide nanodisks with epitaxial alignment on graphene
Source: Nat Commun. 2020 Feb 10;11:823. doi: 10.1038/s41467-020-14481-z (PMC7010709; doi:10.1038/s41467-020-14481-z)
Supplement: Supplementary file 1 — Supplementary Information [file 41467_2020_14481_MOESM1_ESM.pdf]

Atomic Structure and Defect Dynamics of Monolayer Lead Iodide Nanodisks with Epitaxial Alignment on Graphene

Sapna Sinha[1], *Taishan Zhu*[2], *Arthur France-Lanord*[2], *Yuewen Sheng*[1], *Jeffrey C. Grossman*[2], *Kyriakos Porfyrakis*[1], *Jamie H. Warner*[1*]

[1]Department of Materials, University of Oxford, 16 Parks Road, Oxford, OX1 3PH, United Kingdom

[2]Department of Materials Science and Engineering, Massachusetts Institute of Technology, 77 Massachusetts Avenue, Cambridge, MA 02139, USA

Email: *jamie.warner@materials.ox.ac.uk;

Supporting Information

Supplementary Note 1. Sonication duration and stability of dispersed solution

We observed that sonication time is important for the exfoliation of PbI_2. Less sonication leaves multilayer PbI_2 dispersed in the solution whereas longer sonication time renders very small flakes or completely destroys the sheets. Moreover, the dispersed solution was found to be stable for even after a week of the sonication process.

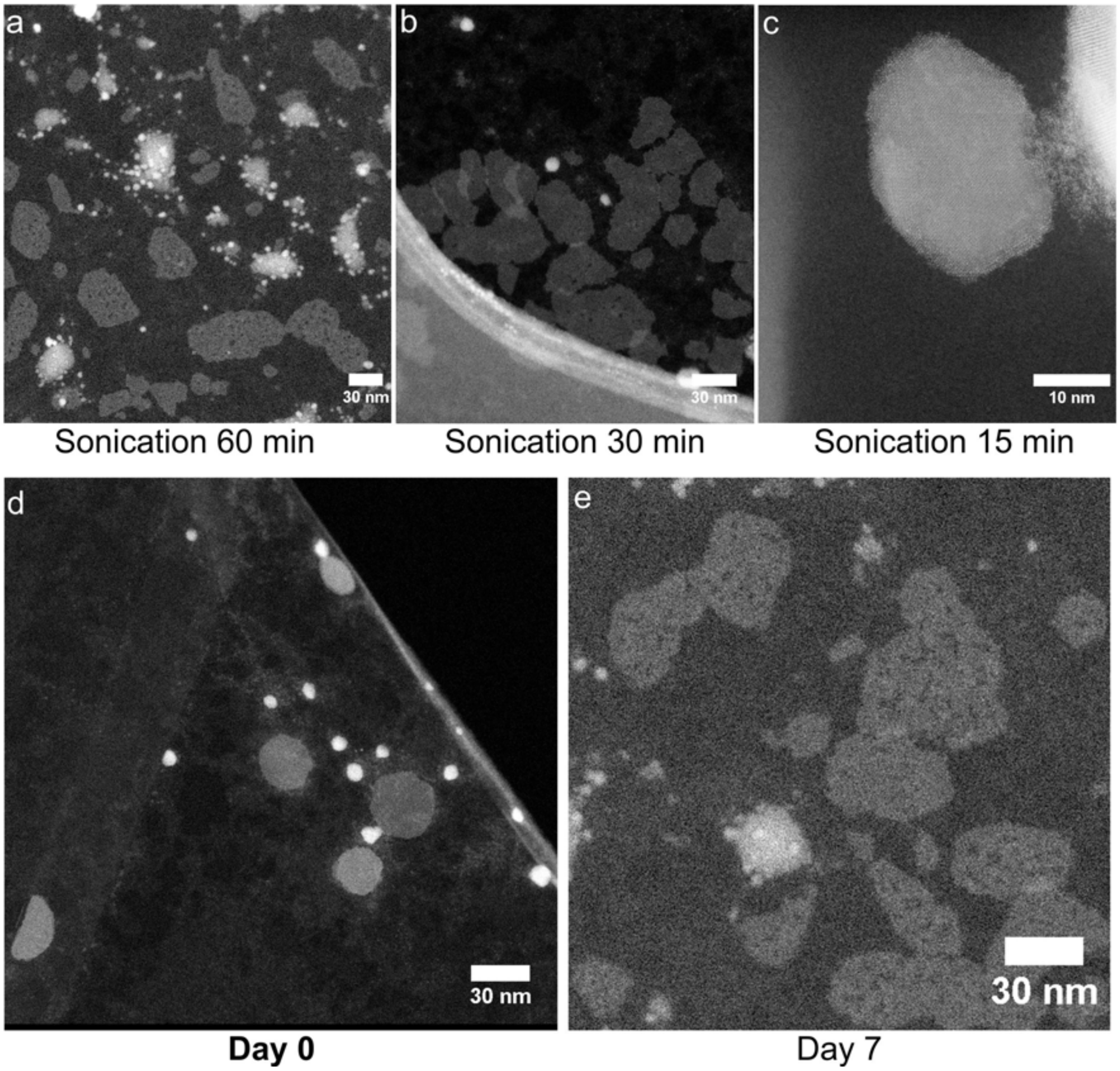

Supplementary Figure 1. ADF-STEM image of PbI_2 suspended on graphene. The exfoliation was carried out via sonication after (a) 60 minutes (b) 30 minutes (c) 15 minutes.

ADF-STEM images of 30-minute sonicated samples on (d) day 0 (a) day 7. Small high contrast nanoparticles are residue from the graphene support and are not from the PbI_2 sample

Supplementary Note 2. Solution used for dropcasting

We have used the top of the supernatant from the dispersed solution to prepare the TEM samples. The thicker PbI_2 settled at the bottom of the container, as shown in supplementary figure 2(a). The thickness of PbI_2 at the bottom and top is not the same. The flakes at the bottom were comparative to the thickness of the flakes that were found at the top of an inadequately sonicated solution (as discussed in Supplementary Note 1).

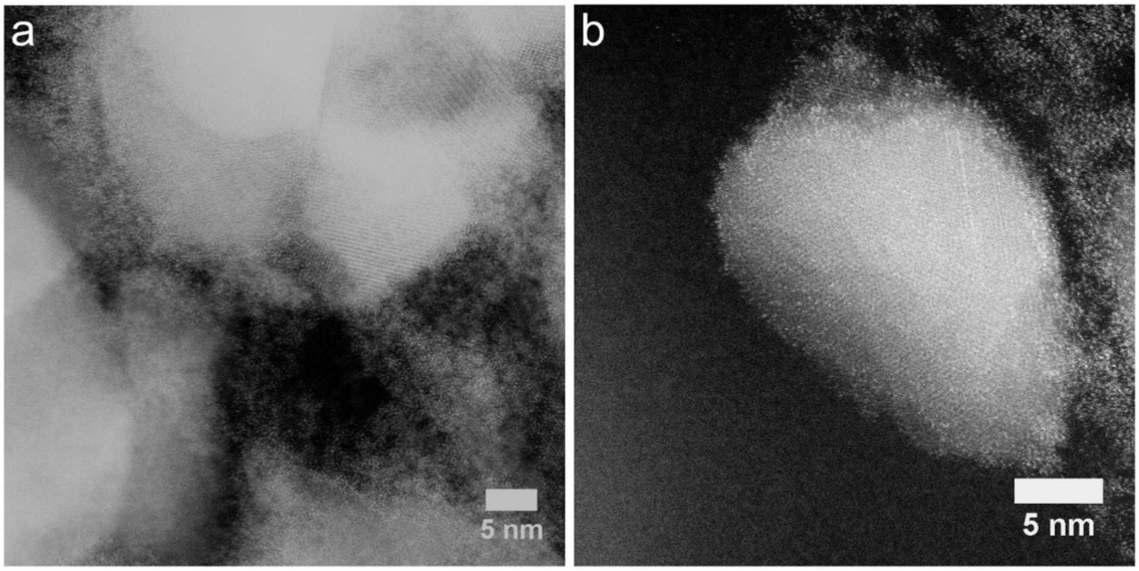

Supplementary Figure 2. ADF-STEM image of PbI_2 suspended on graphene. The exfoliation was carried out via sonication after (a) 30 minutes and the solution was taken from the bottom of the container. (b) 15 minutes and the solution was taken from the top of the container.

Supplementary Note 3. One of the two bilayer PbI_2 found in the experiment.

Supplementary figure 3 shows ADF-STEM images of two flakes of PbI_2 suspended on graphene. Supplementary figure 3(b-c) are the magnified images of the monolayer flake in supplementary figure 3(a) in the blue box. Supplementary figure 3(d-e) are the magnified images of the bilayer flake in supplementary figure 3 (a), in the red box. The intensity difference in ADF-STEM images of the monolayer and bilayer regions can be seen clearly in supplementary figure 3 (a). Supplementary figure 3(f) shows the line profile of the bilayer flake at one of the edges which has some monolayer region. The intensity of the bilayer region, as expected, is twice as high as that of the monolayer region.

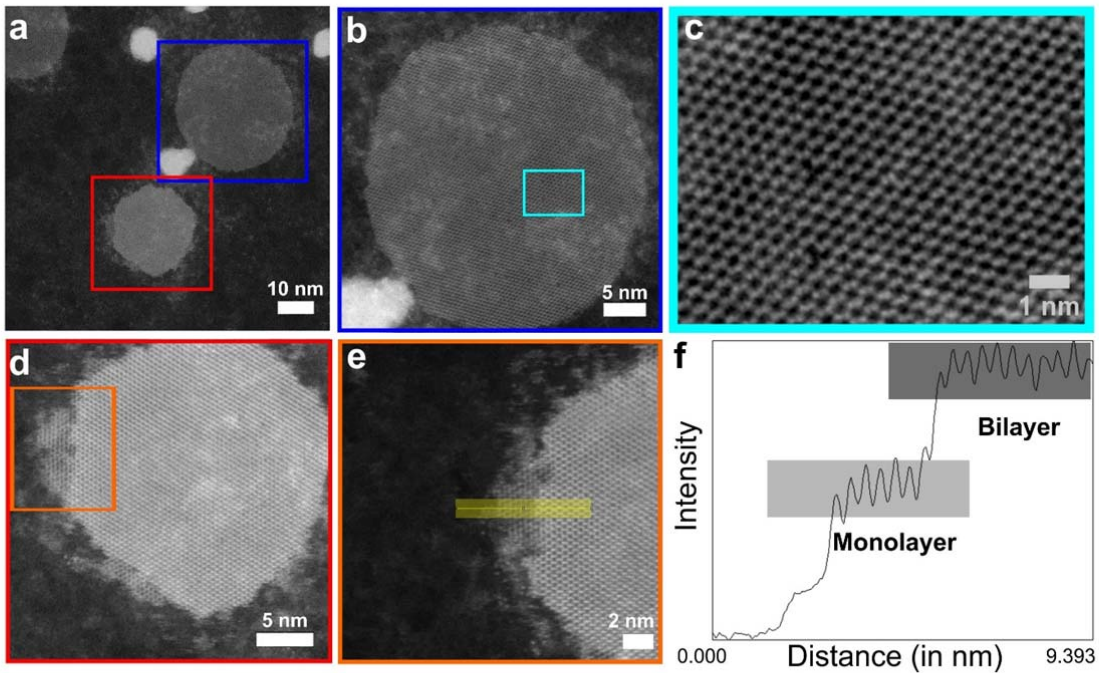

Supplementary Figure 3. (a) Low-magnification ADF-STEM image of monolayer and bilayer regions. (b) High magnification image of monolayer region marked in blue box in panel (a). (c) Higher magnification image of monolayer region marked in cyan colored box in panel (b). (d) High magnification image of bilayer region marked in red box panel (a). (e) Higher magnification image of monolayer and bilayer region in the flake marked in orange colored box in panel (d). (f) Line profile of the region marked with yellow color in panel (e) showing the increase in intensity, confirming the presence of bilayer.

Supplementary Note 4. Nanoparticle residues from graphene sample preparation

The small high contrast nanoparticles come from graphene sample and are not from the PbI_2. supplementary figure4 below shows the graphene samples without any dropcasted PbI_2, suspended on the lacey carbon TEM grid.

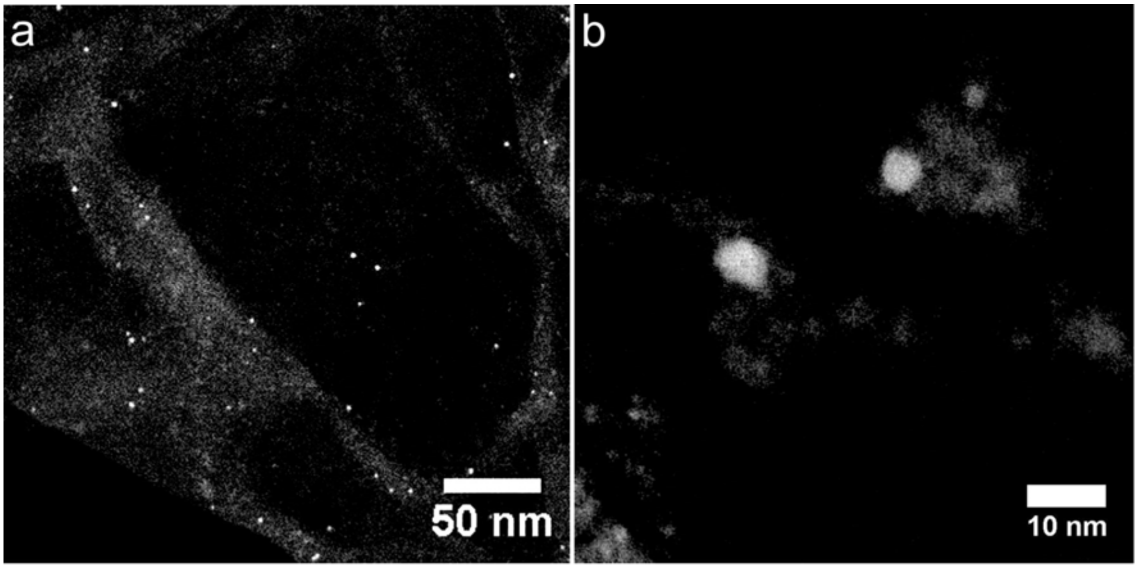

Supplementary Figure 4. (a) Low magnification and (b) High resolution ADF-STEM images of residue nanoparticles on graphene.

Supplementary Note 5. Evidence of monolayer regions instead of bilayer AA’ stacked bilayer PbI_2.

Supplementary figure 5 shows one of the typical flakes of PbI_2 produced by liquid exfoliation. At t=0, the monolayer is as shown in supplementary figure 5(a). However, upon extended exposure from the electron beam, after 2 minutes, there is hole opening up as well as edges damaged. The hole opening can be clearly seen in supplementary figure 5(b), highlighted with red arrow. This indicates that the area in supplementary figure 5(a) is indeed a monolayer instead of AA’ stacked 1H structural phase of lead iodide that looks identical to the monolayer 1H lead iodide. The images are taken at room temperature.

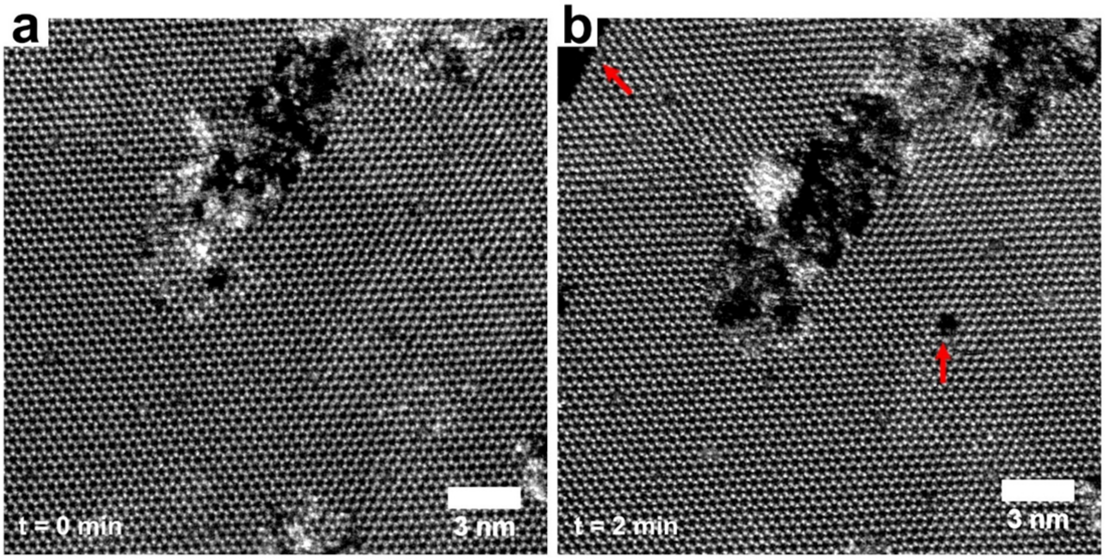

Supplementary Figure 5. (a) ADF STEM image of monlayer lead iodide region at t=0. (c) At t=2min, hole opening up in the monolayer region of supplementary figure a. The red arrows indicate the regions where the defects form.

Supplementary Note 6. All sizes of nanodisks show the 1H structure.

All PbI_2 flakes suspended on graphene, from smallest 3nm to larger hundreds of nanometers in size (supplementary figure 6), showed 1H phase, attributed to epitaxial interactions with the underlying graphene support. The following supplementary figure 6 shows (a) large flake (b) very small flake. The ADF-STEM images here are taken at 60kV.

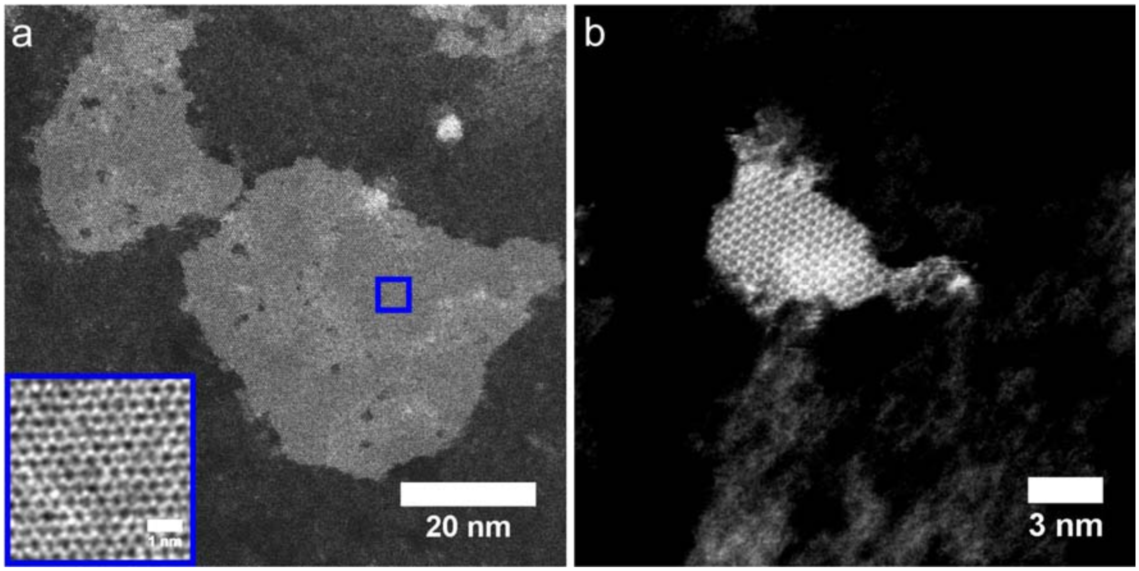

Supplementary Figure 6. ADF-STEM image taken at 60kV of (a) large (b) small PbI_2 suspended on graphene.

Supplementary Note 7. Orientation and lattice direction of PbI_2

Graphene lattice plays a role in influencing the orientation of PbI_2 lattice after it has been drop-casted on top. The CVD grown graphene is known to have grain boundaries and random orientation on the substrate on which it is grown. Hence, different regions of interest showed different PbI_2 orientation but all the PbI_2 crystals in one particular area had the same orientation. supplementary figure 7 shows two different regions on the same sample. Both of them had different orientations because of different graphene domains. However, the flakes in the same region had the same lattice orientation (supplementary figure 7 (a-c); supplementary figure 7 (d-f).

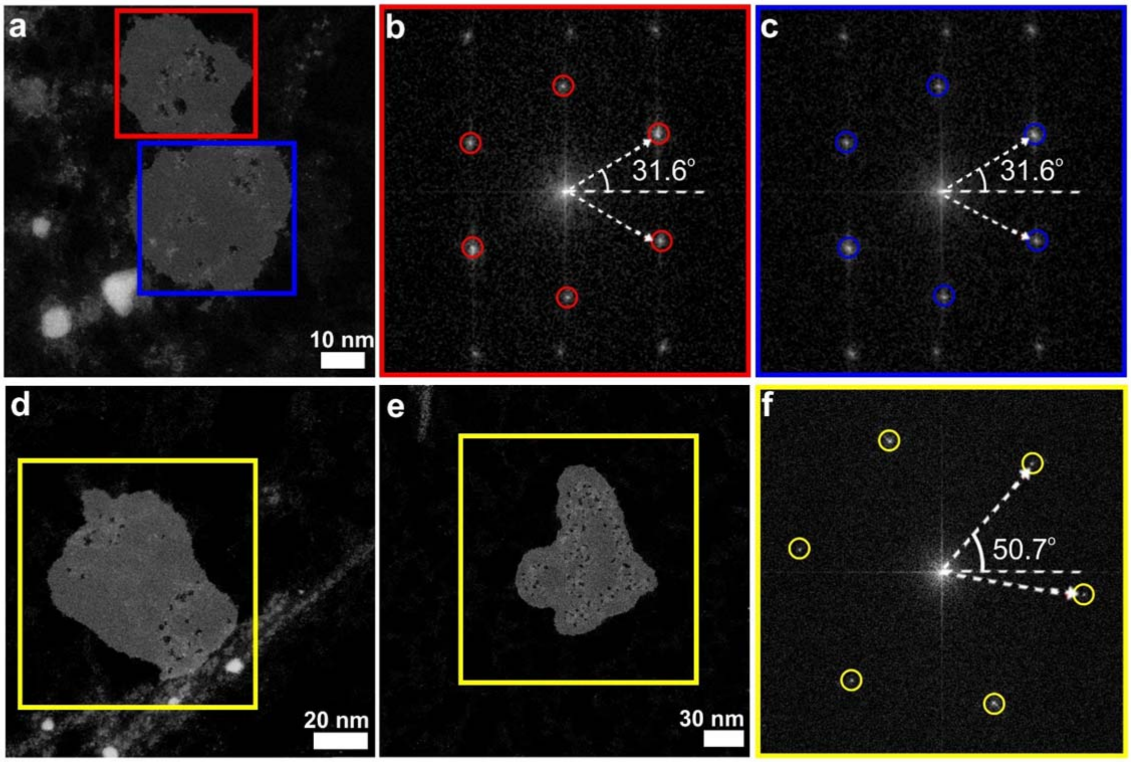

Supplementary Figure 7. (a) ADF-STEM images of two PbI_2 flakes in the same area of interest. (b) FFT of the flake in the red boxed region in supplementary figure a. (c) FFT of the flake in the blue boxed region in supplementary figure a. (d-e) ADF-STEM images of two monolayer crystals of PbI_2 in the same region of interest, close to each other (< 200 nm). (f) FFT of the flakes in the yellow boxed regions of supplementary figure d and e showed the same orientation and pattern.

Supplementary Note 8. Orientation and lattice direction of PbI_2

Further examples of PbI_2 showing the same lattice orientation on top of graphene. All the nanodisks were imaged from the same region of the TEM grids, and hence, all of them orient with similar direction, as it can be seen by their FFT.

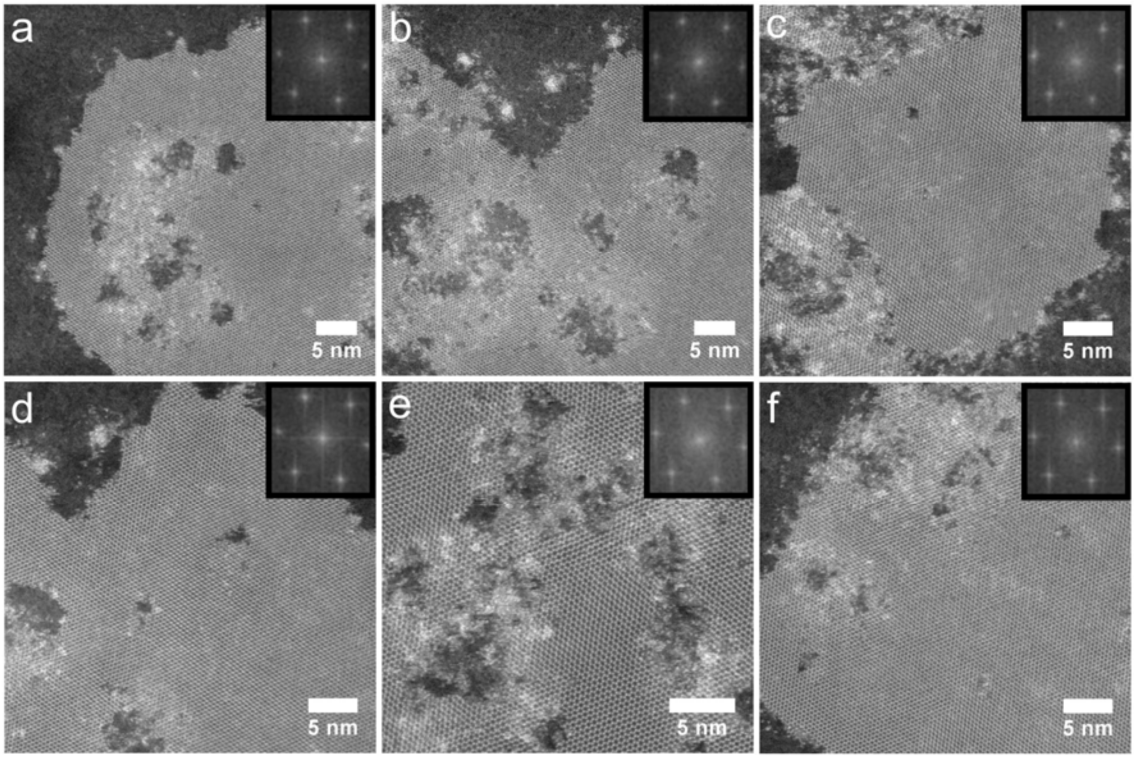

Supplementary Figure 8. (a-f) ADF-STEM images of PbI_2 flakes in the same area of interest. The insets show the FFT of the flake. All the FFTs showed the same orientation and pattern.

Supplementary Note 9. T-phase of lead iodide on lacey carbon grid

PbI_2 flakes on top of lacey carbon grid showed 1T structural phase, an example of which has been shown in the supplementary figure 9. The intensity and the structure of the monolayer flakes are not as good as that of the flakes suspended on grpahene because graphene is a very clean substrate whereas lacey carbon has impurities on top. Intensity of the lead and iodide atoms as well as the inter-column spacing between Pb-Pb, as shown in supplementary figure 9(d) agrees with that of the simulation of 1T phase, discussed in the main text and shown in main text figure 2.

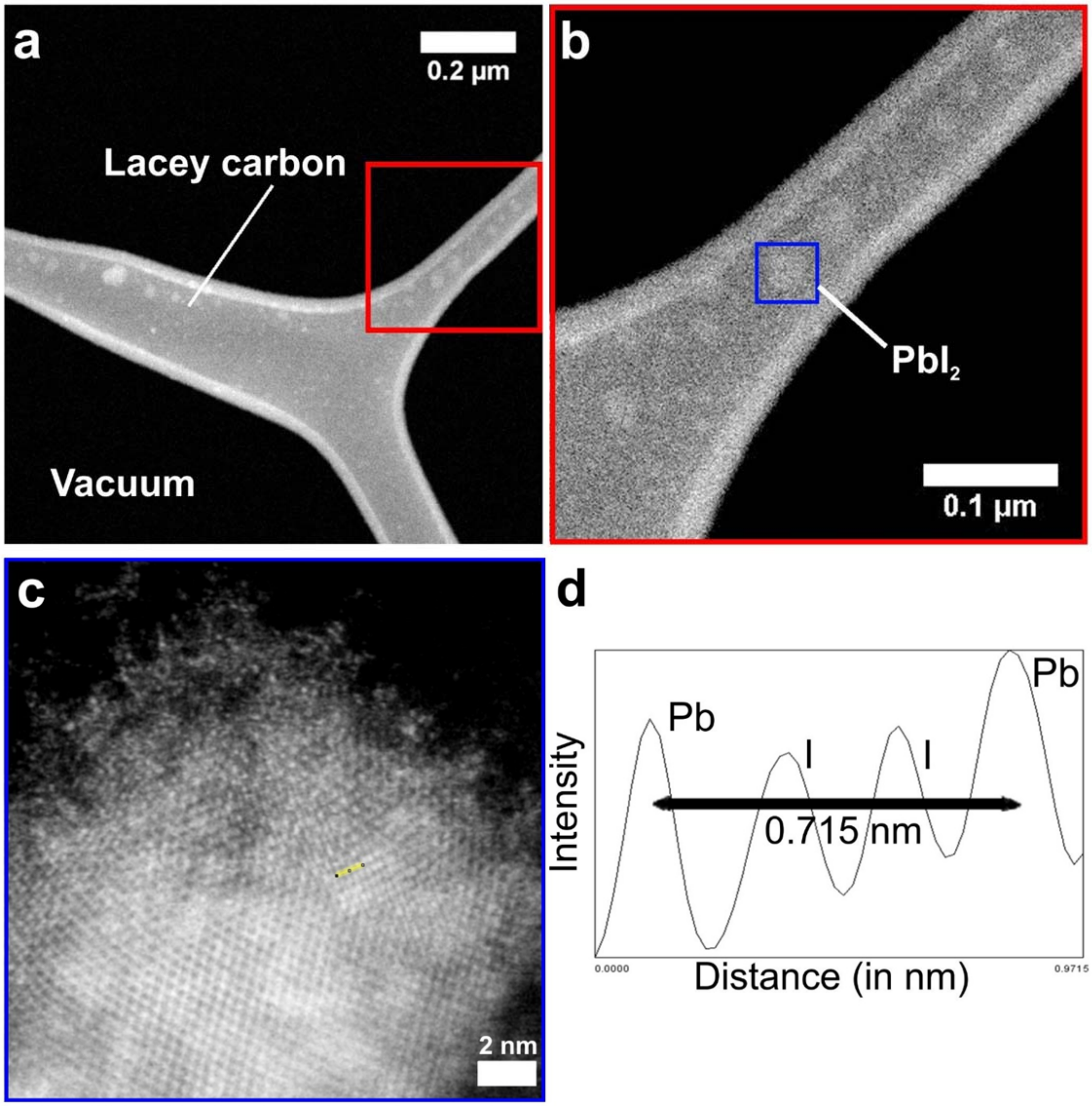

Supplementary Figure 9. (a) Low magnification ADF-STEM image of PbI_2 flakes deposited on top of lacey carbon via the liquid phase exfoliation method. The small white contrasts on the lacey carbon are the PbI_2 flakes. (b) Higher magnification of the region drawn with a red box in (a). (c) Higher magnification of the monolayer region drawn with a blue box in (b). (d) Line profile of the region marked with yellow in (c).

Supplementary Note 10. Transformation of lead iodide flake into 1H structure just after deposition.

ADF-STEM imaging of PbI_2 flakes within 20 minutes after being dropcasted on graphene. We observed mechanical shift in the structure while it transforming into 1H phase. Supplementary figure 10 (a) shows the mixed structural phases in PbI_2 right after dropcasting. The structures other than 1H have been indicated with the X sign. supplementary figure 10 (c) is the high resolution image of the area boxed in red color in supplementary figure 10 (a). An example of line profile from one of the X structural regions is shown in supplementary figure 10 (e). The interatomic distance is very different to that observed for 1T or 1H phases and also differs in different regions, indicating that the material is going some transformations. And after a day, we observed that all the flakes on graphene showed 1H structural phase with same orientations in a given area. Supplementary figure 10 (b) shows the uniform 1H structural phase in PbI_2 after a day. Supplementary figure 10 (d) is the high resolution image of such flake. An example of line profile from any region is shown in supplementary figure 10 (f). The interatomic distance confirms that it is indeed the 1H phase.

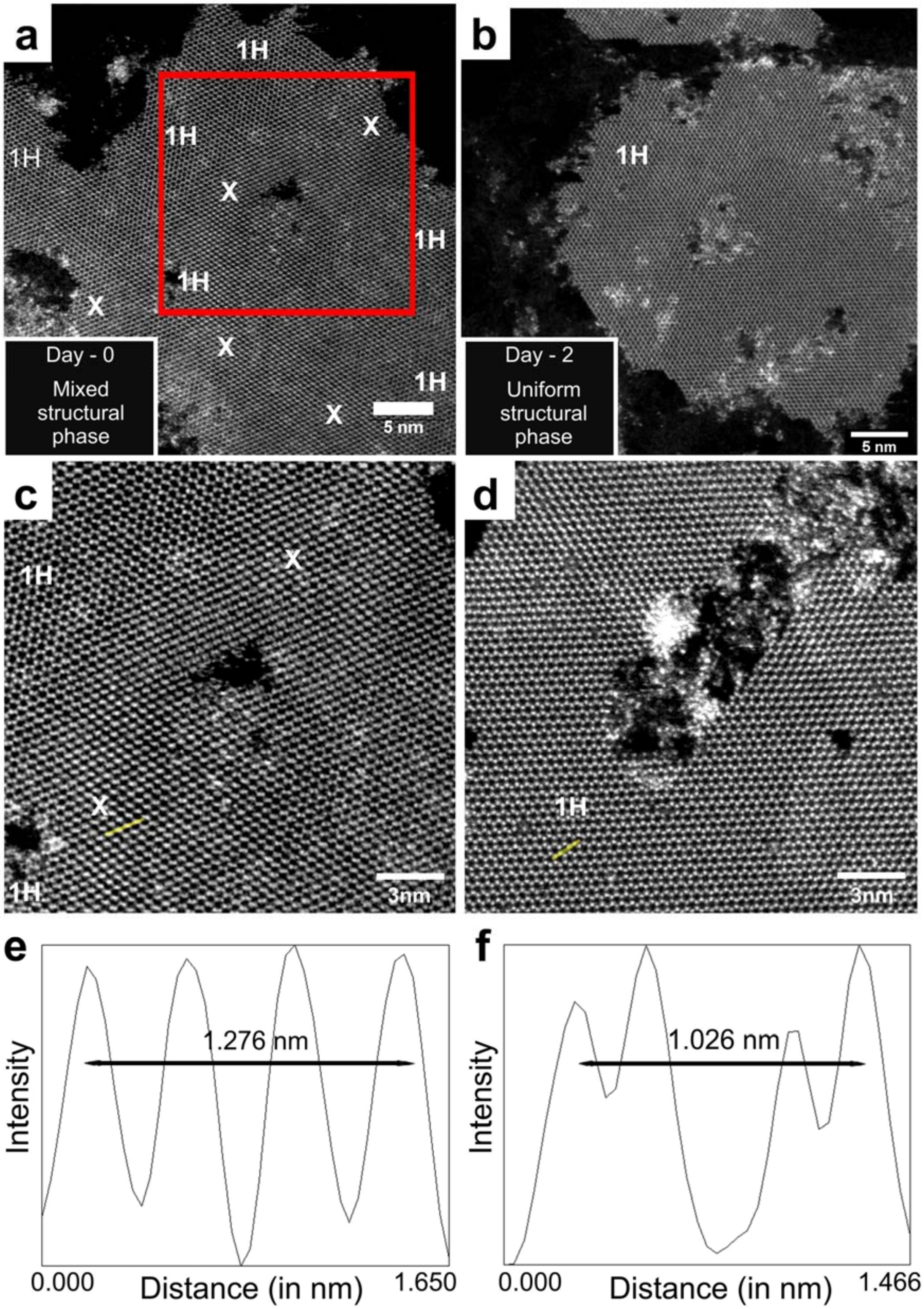

Supplementary Figure 10. (a) ADF-STEM image of mixed structural phases in PbI_2 right after dropcasting. The local regions with stacking other than 1H have been indicated with the X sign. (b) shows the uniform 1H structural phase in PbI_2 after a day. (c) High resolution

image of the area boxed in red color in panel a. (d) High resolution image of uniform 1H structural phase. (e) Line profile from one of the X structural regions is shown in yellow annotation in panel c. (f) Line profile from yellow annotated region in panel d confirming that it is indeed the 1H phase.

Supplementary Note 11. Moire patterns of 1T and 1H on graphene

To understand why PbI_2 adopts 1H structure with concomitant alignment to the armchair direction of graphene, we examine the Moire patterns formed between the two crystals in supplementary figure 11 (a-d). The different lattice spacings of 1T and 1H PbI_2, result in various degrees of commensuration with the underlying graphene lattice. The smaller the difference in the relative lattice spacings between the two crystals, the larger the van der Waals interaction is likely to be. The best lattice match occurs when the PbI_2 adopts 1H phase and is aligned to the arm-chair direction, which agrees with our experimental findings

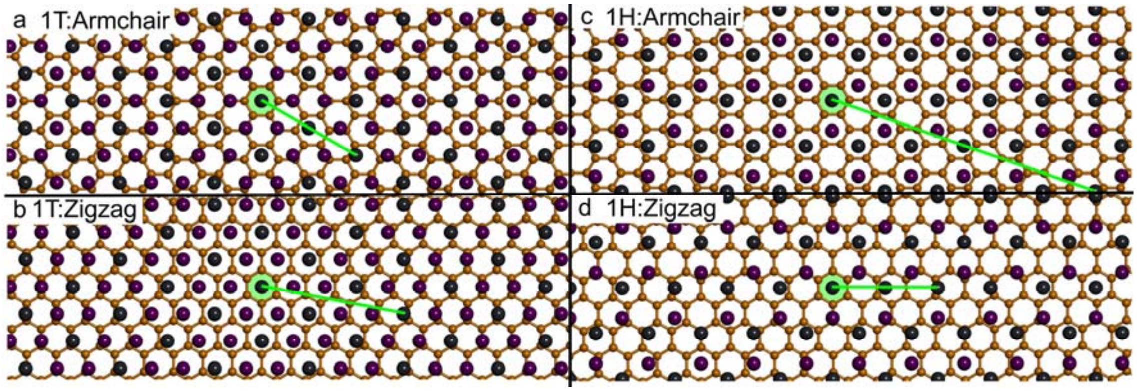

Supplementary Figure 11. Atomic images of (a) PbI_2 (1T) aligned to graphene armchair, (b) PbI_2 (1T) aligned to graphene zigzag, (c) PbI_2 (1H) aligned to graphene armchair, (d) PbI_2 (1H) aligned to graphene zigzag. Green shading indicates the position of Pb located in center of graphene hexagon. Green line indicates the minimum distance where Pb atom overlaps a C-C bond in graphene, indicating loss of commensuration.

Supplementary Note 12. Zigzag PbI_2 edge alignment with Graphene after edge etching

When the edges of PbI_2 are etched to form sharp zig-zag faceted terminations after electron beam irradiation, it maintains alignment with graphene, i.e. the etching takes place in zigzag-PbI_2 and armchair graphene direction. Supplementary figure 12(a) shows the low magnification image of PbI_2 flake on top of graphene at t=0s. Supplementary figure 12(b-c) show the red and blue boxed regions of supplementary figure 12(a) respectively. From these figures, we can see that initially the zigzag PbI_2 was aligned in the armchair graphene direction. Supplementary figure 12(d-e) show the time lapse series of ADF-STEM images recorded after 30 seconds of electron beam exposure. The yellow lines marked in supplementary figure 12(e) show the edge etching directions. Supplementary figure12(f) is the atomic model of the PbI_2/graphene with same lattice orientation as of supplementary figure 12(a). As it can be seen, the FFT of the atomic model is shown in the inset of supplementary figure 12(f) as the PbI_2/graphene nanodisk imaged at t=0s in supplementary figure 12(a). Comparing that with the atomic model of PbI_2/graphene in supplementary figure 12(f), we can see that the termination of the edges in supplementary figure 12(d-e) still conform to the same directionality, i.e. zigzag PbI_2-armchair graphene.

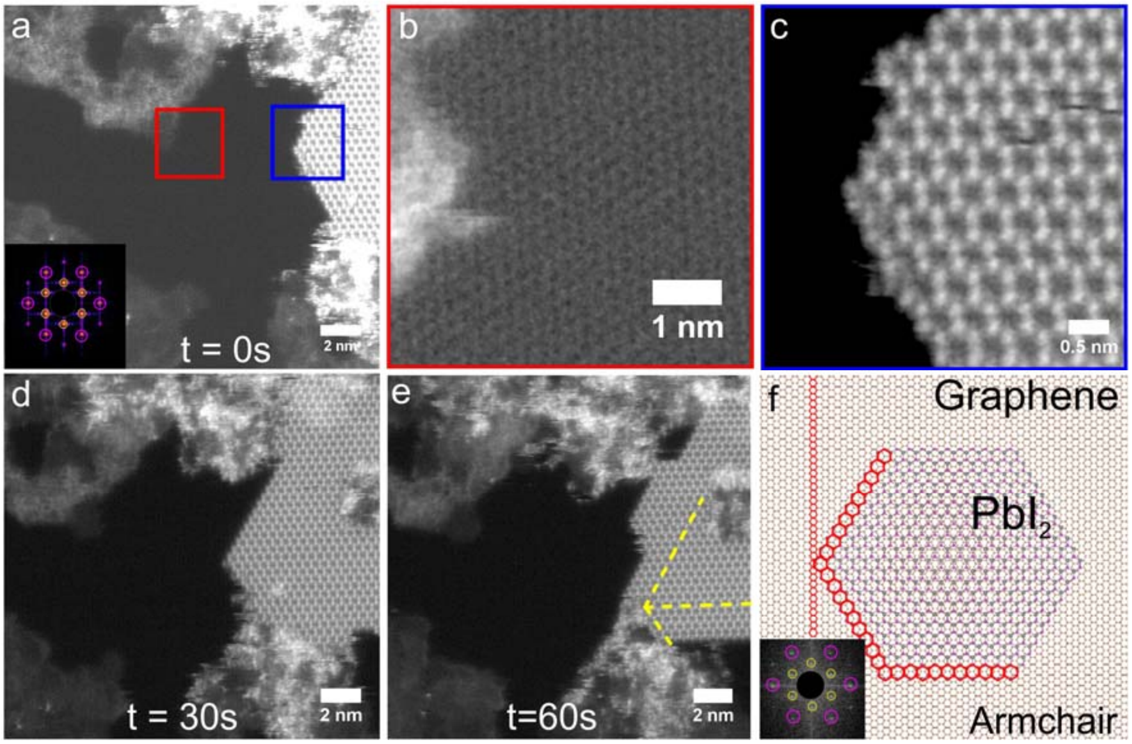

Supplementary Figure 12. (a) ADF-STEM image of PbI_2 flake on top of graphene at t=0s. (b) Higher magnification of the region indicated by the red box in (a) showing the graphene resolution. (c) Higher magnification of the region indicated by the blue box in (a) showing the PbI_2 orientation. (d-e) Time lapse ADF-STEM image of the edge at t = 0 seconds and t = 30 seconds of electron beam exposure. The yellow arrow indicates the ejection of the atoms from the edges due to the damage from the electron beam, leading to ‘un-zipping’ of the chain, maintaining the zig-zag edge of the flakes. (f) Atomic model of the PbI_2/graphene with same lattice orientation as of supplementary figure (a). The Inset shows the FFT of the atomic model.

Supplementary Note 13. Relative alignment of time frame of PbI_2 etching

Following supplementary figure 13 shows the time lapse ADF-STEM image for the relative alignment frames for the region of PbI_2 shown in main text figure 4(j-l). Supplementary figure13(a-c) shows the relative alignment of the picture frame based on the presence of the stable defect shown in red and blue boxes (higher resolution images shown in the supplementary figure 13(d-f) and also on the accumulated atomic clusters shown by the orange and yellow arrowheads. The following figures show the relative alignment of the time frames of the captured images shown in figure 4(j-l) of the main text.

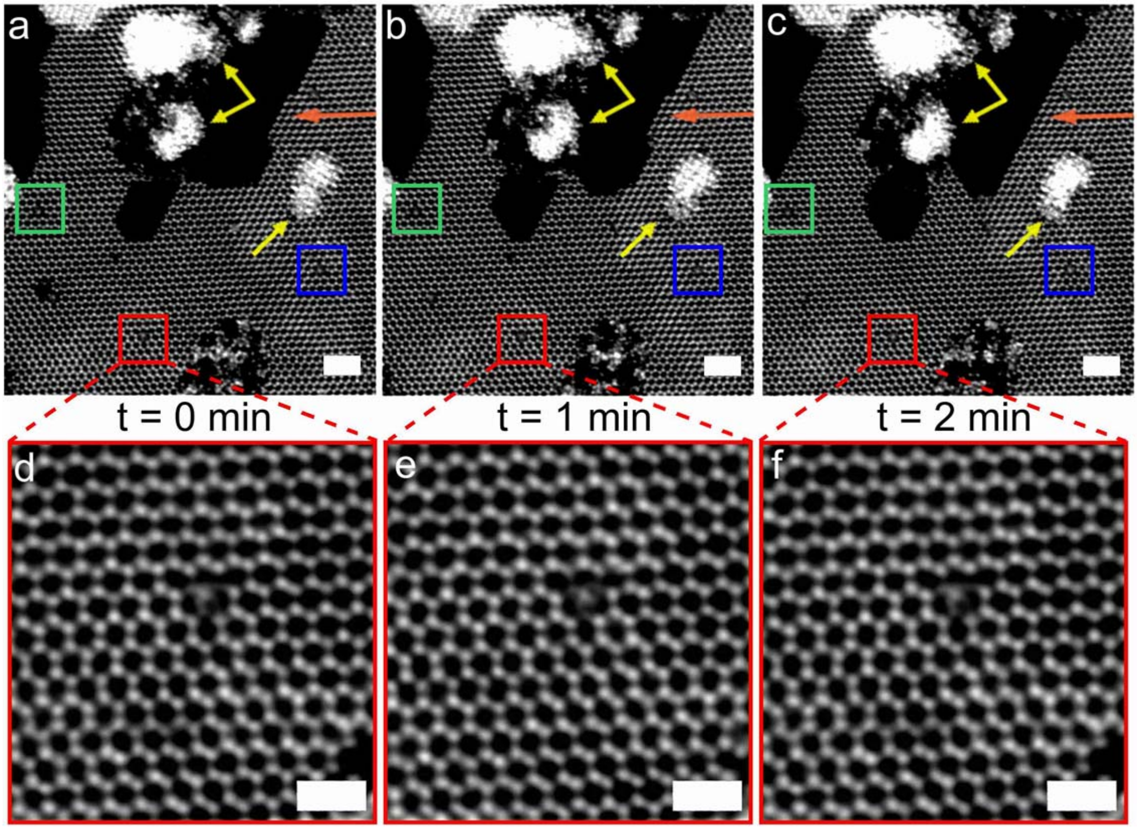

Supplementary Figure 13. (a-c) ADF-STEM time lapse image of a section of PbI_2 taken after every 1 minute of electron beam irradiation. The red and blue boxes show the stable defect and yellow and orange arrowhead are used to point the accumulated atomic clusters which have been used for relative alignment of the frames. (d-f) Higher resolution images of the stable defect boxed in red in panels (a-c) respectively.

Supplementary Note 14. Process of determining some defects

The interpretation and assignment of the experimental images to their simulated configurations, in figure 5 of the main text, were made on the line profile of the defects after normalizing their intensities. As it can be seen, the line profiles of the corresponding images, in the following supplementary figures 14(a-d), show clear Lead and Iodide vacancies in blue and red respectively. The supplementary figure 14(d) shows the line profile shows 2I + 2I vacancy, but also, we can deduce that there is also 1Pb vacancy, as we are unable to see the nearest neighbor contrast from the Pb atom which is prominent in the supplementary figure 14(c).

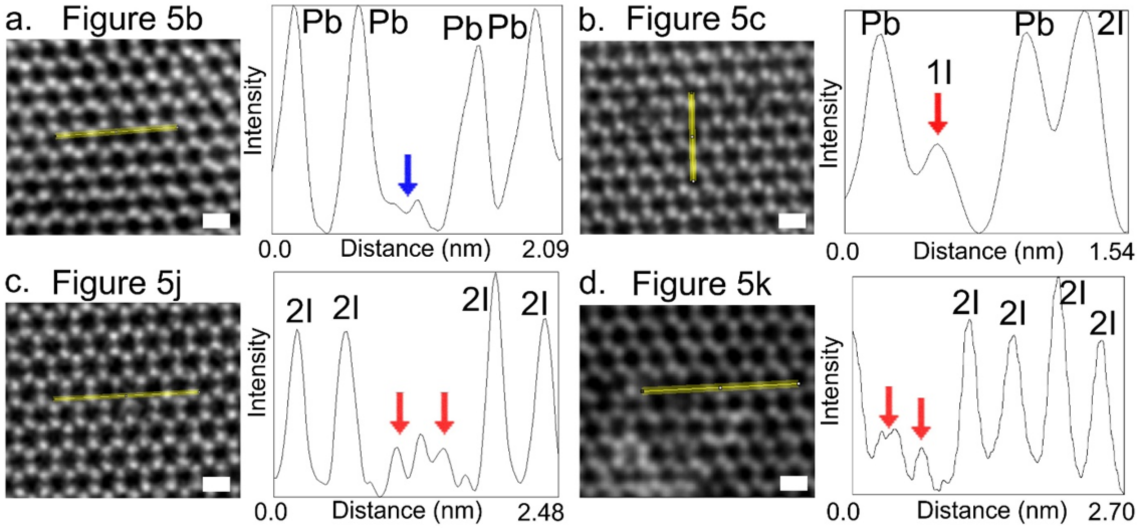

Supplementary Figure 14. ADF-STEM image of lead iodide defects. The blue and red arrows correspond to lead and iodide vacancies respectively. (a) 1 Lead vacancy, as can be seen from corresponding line profile. (b) 1 Iodide vacancy, as can be seen from the corresponding line profile. (c) 2I + 2I vacancies, as can be seen from the corresponding line profile. (d) The line profile shows 2I + 2I + 1Pb vacancy, as seen in the corresponding line profile. All scale bars correspond to 0.5 nm.

Supplementary Note 15. Sputtering energy

A 10×10×1 supercell is used for calculating the sputtering energy. The sputtering energy is defined by the energy difference between the crystalline structure and the one with a vacancy, for instance, the I sputtering energy is $\Delta E_I = E_{crystal} - (E_{V_I} + E_I)$. The energy tolerance is set to 0.0001 for all energy calculations. The following supplementary figure 15 shows (a) Pristine supercell, (b) supercell with one I vacancy, and (c) supercell with one Pb vacancy. The energy differences, as calculated are 3.15 eV and 6.36 eV for Iodide and Lead, as shown in the equations (1) and (2) respectively.

$$\Delta E_I = E_{crystal} - \left(E_{V_I} + E_I\right) = (-837.01) - (-840.16) = 3.15\, eV \quad (1)$$

$$\Delta E_{Pb} = E_{crystal} - \left(E_{V_{Pb}} + E_{Pb}\right) = (-833.80) - (-840.16) = 6.36\, eV \quad (2)$$

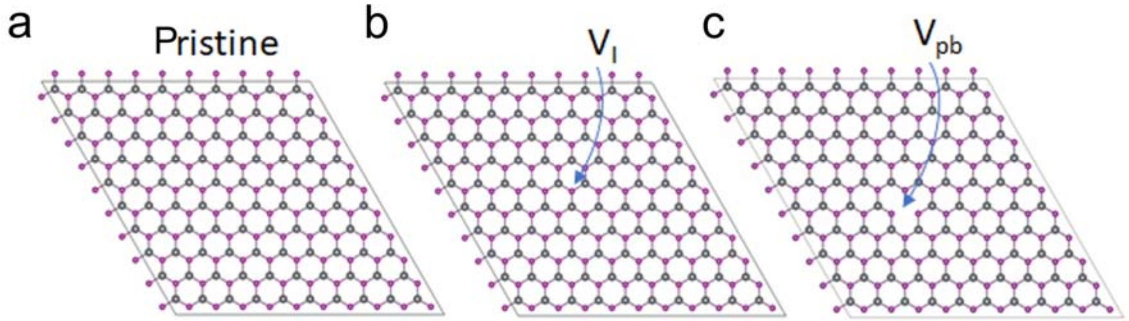

Supplementary Figure 15. Atomic model of the PbI_2 (a) pristine supercell (b) one I vacancy supercell (c) one Pb vacancy.

Supplementary Note 16. Accumulation of lead particles after etching

The following supplementary figure 16 shows the effect of the accumulation of the atoms after electron beam exposure. Iodine atoms are very light and hence, the likelihood of them getting displaced from the lattice is higher. The contrast of the single atoms imaged are of similar magnitude to that of Lead within the PbI_2 lattice. The atoms aggregate to form amorphous clusters, as shown with red arrows in supplementary figure 16(a) or get dispersed on the surface of graphene as shown in supplementary figure 16(b).

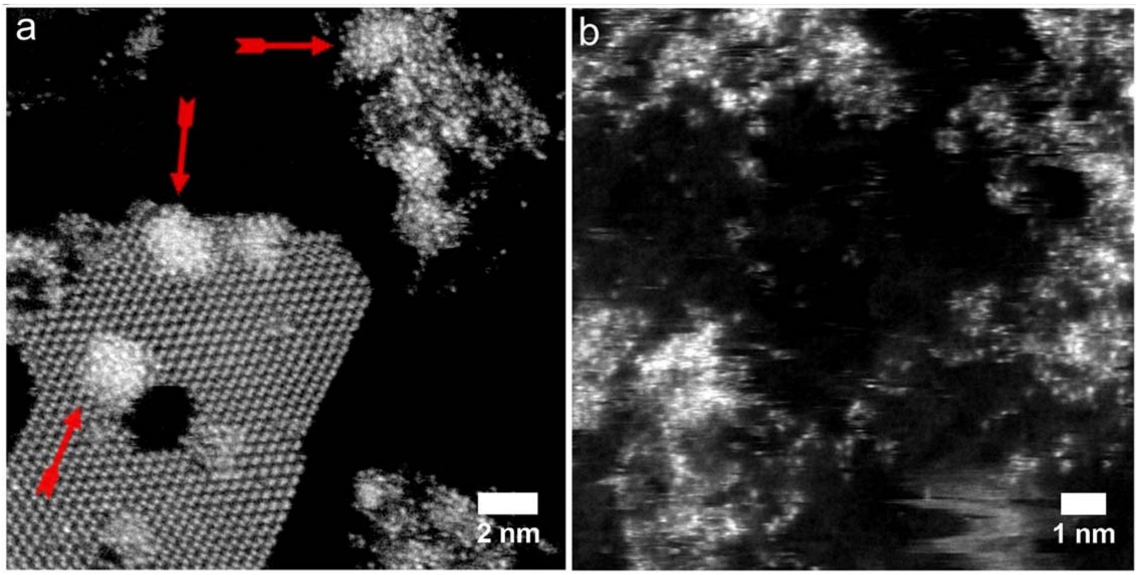

Supplementary Figure 16. (a) ADF-STEM image of lead atoms forming clusters (b) ADF-STEM image of lead forming dispersion on the graphene.

Supplementary Note 17. Vacancy migration

10×10 supercells and 4×4 k-point grids are employed, and a criterion of 0.03 eV/ Å is set for the force convergence. For V_I migration, two pathways are considered: in plane and out-of-plane directions, and only in-plane V_{Pb} migration is considered. The starting and end points for NEB search are prepared by removing the corresponding atoms from the relaxed pristine structure, and then further relaxed. The 6 intermediate images are interpolated by the Vasp TST toolkit. The converged energy paths are the fitted by cubic spline functions.

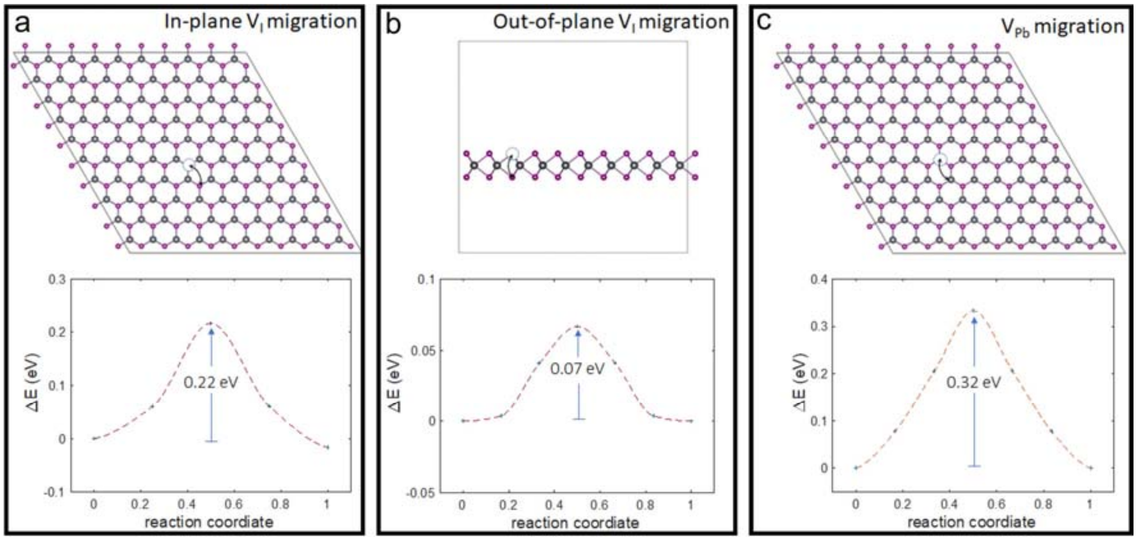

Supplementary Figure 17. Atomic structure and energy profile for vacancy migration of (a) Iodide atom in-plane (b) Iodide atom out-of-plane and (c) Lead atom.
